# Stage II/III Rectal Cancer Post-Treatment Surveillance Patterns of Care: A SEER- Medicare Study

**DOI:** 10.19080/argh.2021.17.555972

**Published:** 2021-09-30

**Authors:** Catherine Chioreso, Mary C Schroeder, Irena Gribovskaja Rupp, Eric Ammann, Knute D Carter, Charles F Lynch, Elizabeth A Chrischilles, Mary E Charlton

**Affiliations:** 1 Department of Epidemiology, University of Iowa College of Public Health, USA; 2 Department of Pharmacy Practice and Science, University of Iowa College of Pharmacy, USA; 3 Department of Surgery, University of Iowa Carver College of Medicine, USA; 4 Department of Biostatistics, University of Iowa College of Public Health, USA; 5 Iowa Cancer Registry, University of Iowa College of Public Health, USA

**Keywords:** Rectal cancer, Follow-up, Surveillance, Post-treatment patterns of care

## Abstract

**Introduction:**

Despite high rectal cancer recurrence rates, knowledge on post-treatment surveillance utilization is limited. Hence, this study aims to estimate patterns of post-treatment surveillance and determine associated factors.

**Patients and Methods:**

Retrospective study of 1,024 SEER-Medicare patients >65 years old diagnosed with stage II/III rectal cancer between 2007-2013. Logistic regression was used to determine factors associated with ≥1 colonoscopy, ≥2 physician visits, ≥2 carcinoembryonic antigen (CEA) tests and ≥2 computed tomographic colonography (CT) within 14 months after primary treatment.

**Results:**

Fifty-five percent had ≥1 colonoscopy, 54% had ≥2 physician visits, 47% had ≥2 CEA tests and 20% had ≥2 CTs. In multivariable logistic models, younger age and receipt of chemoradiation therapy (vs none) were significant across all surveillance procedures while clinical factors such as comorbidity were not. Being married (OR=1.69; 95% CI: 1.26-2.26) and proximity to a high-volume hospital (≤15 vs >30 minutes, OR=1.56; 95% CI: 1.00-2.43) were associated with ≥1 colonoscopy. Female gender (OR=1.56; 95% CI: 1.17-2.09), being married (OR=1.56; 95% CI: 1.17-2.08), white race (OR=1.79; 95% CI: 1.23- 2.62) and surgery from high-volume surgeon (OR=1.47; 95% CI: 1.06-2.04) were associated with ≥2 physician visits. Female gender (OR=1.45; 95% CI: 1.08-1.95), being married (OR=1.46; 95% CI: 1.08-1.96) and surgery from high-volume surgeon (OR=1.55; 95% CI: 1.10-2.17) had higher ≥2 CEA tests.

**Conclusions:**

Post-treatment surveillance remains low but is more common among younger patients and recipients of chemoradiation. Distinct profiles of patient characteristics and provider volume were associated with individual surveillance procedures suggesting the need for multicomponent strategies to increase surveillance.

## Introduction

Approximately 45,000 rectal cancer cases in the US are expected in 2021 [1]. Advances in rectal cancer management, such as total mesorectal excision (TME) and neoadjuvant therapy, have been associated with reduced recurrences and better survival outcomes [2,3]. Despite these rectal cancer management advances, 5-year survival rates average 64% mostly due to high stage II/III rectal cancer recurrence rates (≈ 40%) [4,5]. Previous research has suggested that early asymptomatic recurrence detection via post-treatment surveillance doubles the odds of receiving curative surgery [6] and can ultimately improve survival outcomes [4,7-9].

Between 2007 and 2020, the National Comprehensive Cancer Network (NCCN) recommended post-treatment colonoscopy one year after primary rectal cancer therapy while physician visits, carcinoembryonic antigen (CEA) tests and pelvic computed tomographic colonography (CT) were recommended every 3-6 months in the first two years after primary rectal cancer therapy [10]. Previous studies have reported inconsistent colorectal cancer post-treatment surveillance uptake [11-15]. and there is limited knowledge on factors associated with receipt of stage II/III rectal cancer surveillance. Therefore, the objective of this study is to determine utilization rates and factors associated with post-treatment colonoscopy, physician visits, CEA tests and CTs for stage II/III rectal cancer patients.

## Materials and Methods

### Data sources

The University of Iowa Institutional Review Board approved this Surveillance, Epidemiology and End Results (SEER)- Medicare retrospective cohort study. SEER data contain demographic, tumor, cancer treatment and survival information from 18 population-based cancer registries representing approximately 28% of the US [16,17]. Medicare data contain diagnoses and procedure information for 94% of the US population aged ≥65 years.17 The National Cancer Institute (NCI) and Centers for Medicare and Medicaid Services (CMS) link SEER and Medicare data by date of birth, social security number and gender.

### Study population

**Figure 1:**
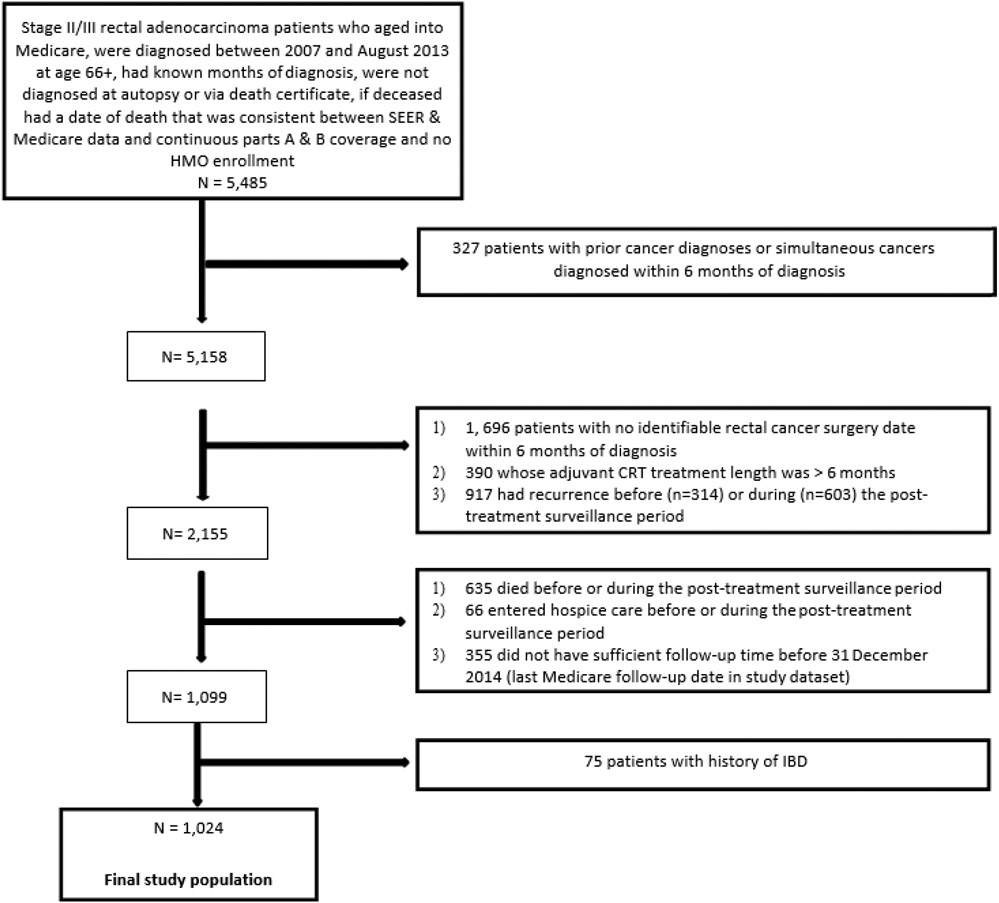
Flow chart of study population.

The study population (Figure 1) met the following inclusion criteria:

- primary stage II/III rectal (ICD-O-3 site: C209) adenocarcinoma (histology: 8140-8571) patients who aged into Medicare and were not diagnosed via autopsy or death certificate between January 2007 and August 2013 at age 66+
- no simultaneous cancer diagnosed within 6 months of diagnosis
- identifiable Medicare rectal cancer surgery date within 6 months of diagnosis
- consistent date of death between SEER and Medicare files
- continuous Parts A and B Medicare coverage and no HMO coverage during study period to enable complete healthcare utilization assessment
- no history of inflammatory bowel disease
- sufficient 14-month follow-up time before 31 December

2014 (last Medicare follow-up date in study dataset). Since patients at the end of life have different patterns of care, those who were admitted into hospice care or died before or during the post-treatment surveillance period were excluded [12].

Patients who had a recurrence before the post-treatment surveillance period (n=314) were excluded since they were ineligible for surveillance. Patients who had recurrence during the surveillance period (n=603) were excluded to ensure the identification of surveillance-related procedures [11,12,18]. Recurrent cancer diagnosis and treatment after primary cancer surgery were derived using International Classification of Diseases, Ninth Revision Clinical Modification (ICD-9) diagnosis and Current Procedural Terminology, Fourth Edition (CPT) codes (Appendix Table A1) [19]. Recurrent cancer surgery was identified >90 days after surgery to factor in surgical complications. If a patient had surgery but no adjuvant therapy, chemotherapy or radiotherapy received >120 days after surgery was identified as recurrent treatment. Among patients who had surgery and adjuvant therapy, chemotherapy or radiotherapy received >90 days after last adjuvant therapy claim was considered recurrent treatment. The date of recurrence was identified as the minimum date of recurrence diagnosis or treatment.19 Patients whose adjuvant treatment was >6 months (i.e., start date to end date) were excluded since it could be indicative of recurrence.

### Rectal cancer treatment and post-treatment surveillance

Rectal cancer surgery was derived from CPT and ICD-9 codes (Appendix Table A1) [20]. Medicare CPT and ICD-9 codes and their respective dates were used to derive chemoradiation therapy (CRT) sequence (i.e., no CRT, neoadjuvant CRT plus adjuvant chemotherapy, neoadjuvant CRT, and adjuvant CRT); adjuvant therapy had to be within 120 days of primary surgery treatment. The last date of treatment for patients who did not receive adjuvant therapy was date of surgery (Figure 2a), but for those who received adjuvant therapy, it was the last adjuvant chemotherapy or radiotherapy claim (Figure 2b), The post-treatment surveillance period began 30 days after the last rectal cancer primary treatment date (i.e., surgery, chemotherapy or radiation). Patients were observed for 14 months after primary rectal cancer treatment to allow for scheduling delays encountered in real-world clinical care.

CPT and ICD-9 codes were used to identify the frequency and time to first post-treatment colonoscopy (CPT: 44388-44389, 44392-44394, 44397, 45100, 45108, 45300, 45303, 45305, 45307-45309, 45315, 45317, 45320-45321, 45327, 45330-45335, 45337-45342, 45345, 45355, 45378-45387, 45391-45392, 45382-45385, 45499, 45990, 45999, 74261-74263, 74270, 74280, 82270-82272, 82274, 10021-10022, G0104-G0107, G01020, G0122, G0328, G0464; ICD-9: 45.21-45.29, 45.41-45.43, 48.21-48.26, 48.29, 54.11, 89.34), physician visits to oncologist, primary care provider and surgeon specialties that typically perform surveillance (CPT: 99201-99245, 99381-99397; ICD-9: V70, V70.0, V70.9, V72, V72.9), CEA test (CPT: 82378) and CT (CPT: 71250, 71260, 71270, 72191-72194, 74150, 74160, 74170, 71275, 74175-74178, 75635) [11-15]. Physician specialty was derived from National Claims History (NCH) and American Medical Association files. Subsequent procedures were counted if they occurred >90 days after prior procedure to avoid over-estimating surveillance (e.g., colonoscopy redo due to incomplete bowel preparation) [14]. Colonoscopies with ICD-9 codes for symptoms, such as gastrointestinal bleeding and anemia (Appendix Table A1), were considered ‘indicated’ (vs. for surveillance purposes only) [21].

### Patient characteristics

Patient age, gender, marital status, race and AJCC 6^th^ edition stage were extracted from SEER Patient Entitlement and Diagnosis Summary File (PEDSF). PEDSF patient ZIP code was used to classify rural status using the 2006 Rural–Urban Commuting Area (RUCA) classification system [22]. Percent living below the federal poverty level and percent with at least a 4- year college education from the Tract census file were dichotomized by median percentage [23]. A one-year Medicare claims lookback period was used to derive Charlson comorbidity scores and Function-Related Indicators (FRIs). The Charlson score is an established predictor of one-year survival [24]. FRIs reflect diminished functional capacity based on diagnosis codes for conditions such as dementia, malnutrition and home oxygen use [25].

Surgical complications, such as surgical site infection, were defined using Hendren et al.’s algorithm [26]. Hospital and surgeon rectal cancer surgery volume was defined as the sum of 2007-2013 SEER-Medicare rectal cancer surgeries within 6 months of diagnosis [20]. this correlates well with total volume [27,28] Hospitals (≥14 surgeries) and surgeons (≥5 surgeries) in the fourth volume quartile were considered high-volume hospitals (HVH) and high-volume surgeons (HVS), respectively. As published previously, NCI designated comprehensive centers were classified as HVHs because they are an indicator of quality cancer care while colorectal cancer surgeons or surgical oncologists were considered HVSs due to their specialized training [20]. Travel time between the centroids of patient residence and the nearest HVH ZIP code were calculated [29] and used as a proxy for access to care [30].

### Statistical analysis

The 4 individual measures of post-treatment surveillance (≥1 colonoscopy, ≥2 physician visits, ≥2 CEA tests, and ≥2 CTs) were the outcomes of interest. The Chi-square test was used to determine variation in surveillance uptake by patient characteristics. Multivariable logistic regression was used to ascertain factors associated with surveillance. In four separate sensitivity analyses we

- excluded patients with ‘indicated’ colonoscopies to account for potential reason for procedure
- restricted physician visits to oncologists and colorectal surgeon specialists to test robustness of results for visits to cancer physician specialists
- included recurrent patients in the analysis and considered them adherent on the basis that the goal of surveillance is to detect recurrence to estimate potential maximum surveillance rates
- excluded patients aged 80+ who tend to forgo recurrence treatment to determine extent of recurrence misclassification on surveillance rates [16,19].

## Results

### Overall study population

The median age of the eligible 1,024 patients with stage II/III rectal cancer was 77 (IQR: 71-82). Most patients were white (86%) and lived in urban areas (78%). Half of the patients were married. Forty-seven percent had a Charlson score ≥1 and 35% had ≥1 function-related indicator (indicative of functional impairment]. Fifty-two percent of patients had neoadjuvant CRT (with or without adjuvant therapy), 8% had adjuvant CRT and 25% had no CRT. More than sixty percent of patients had surgery from HVS (62%) and HVH (62%).

### Colonoscopy

As Table 1 shows, 560 (55%) patients received ≥1 colonoscopies. The median months from the end of treatment to first colonoscopy were 5 (IQR: 3-9). The median number of colonoscopies for the entire cohort and for patients who had ≥lcolonoscopies was 1 (IQR=0-1) and 1 (IQR=1-2), respectively. In bivariate analyses, younger age, being married, lower comorbidity, lower function-related indicator, any CRT sequence (vs none), not experiencing surgical complications, surgery from HVS, surgery from HVH, and proximity to HVH were associated with having ≥1 colonoscopy (Table 2).

In multivariable analysis, younger age (>66-70 vs > 80 years, OR=3.23; 95% CI: 2.18-4.78; >70-75 vs > 80 years, OR=2.48; 95% CI: 1.70-3.62; >75-80 vs > 80 years, OR=2.58; 95% CI: 1.77-3.77), being married (OR=1.69; 95% CI: 1.26-2.26) and residing closer to a HVH (≤15 vs >30 minutes, OR=1.56; 95% CI: 1.00-2.43) were associated with receiving ≥1 colonoscopy (Table 3), Compared to no CRT, receiving adjuvant CRT (OR=3.97; 95% CI: 2.11-7.47) and neoadjuvant CRT plus adjuvant chemotherapy (OR=1.90; 95% CI: 1.27-2.85) were associated with having ≥1 colonoscopy.

### Physician visit

As Table 1 shows, 552 (54%) patients received ≥2 physician visits (85% had ≥1). The median months to first physician visit was 2 (1-4). The median number of physician visits for all patients was 2 (IQR=1-2) and 2 (IQR=2-3) for those who had ≥2 physician visits. In bivariate analyses, having ≥2 physician visits was associated with younger age, being married, white race, living below the federal poverty indicator level, lower comorbidity, lower function-related indicator, any CRT sequence (vs none), not experiencing surgical complications, and surgery from HVS (Table 2).

As shown in Table 3, younger age (>70-75 vs > 80 years, OR=1.61; 95% CI: 1.10-2.34; >75-80 vs > 80 years, OR=1.57; 95% CI: 1.08-2.28), female gender (OR=1.56; 95% CI: 1.17-2.09), being married (OR=1.56; 95% CI: 1.17-2.08), white race (OR=1.79; 95% CI: 1.23-2.62) and surgery from HVS (OR=1.47; 95% CI: 1.06-2.04) were associated with higher odds of receiving ≥2 physician visits. Compared to no CRT, receiving neoadjuvant CRT plus adjuvant chemotherapy (OR=2.71; 95% CI: 1.81-4.06), neoadjuvant CRT (OR=1.70; 95% CI: 1.17-2.45) and adjuvant CRT (OR=2.41; 95% CI: 1.37-4.24) were associated with ≥2 physician visits.

### CEA tests

Forty-seven percent (n=478) of the cohort had ≥2 CEA tests; 66% had ≥1 CEA test (Table 1). The median months to first CEA test was 4 (IQR=2-6). The median number of CEA tests for the entire cohort and patients who had ≥2 CEA tests was 1 (IQR=0-2) and 3 (IQR=2-3), respectively. In bivariate analyses, receiving ≥2 CEA tests was associated with younger age, being married, lower comorbidity, lower function-related indicator, any CRT sequence (vs none), not experiencing surgical complications, surgery from HVS and surgery from HVH (Table 2).

Younger age (OR=1.81; 95% CI: 1.23-2.66), female gender (OR=1.45; 95% CI: 1.08-1.95), being married (OR=1.46; 95% CI: 1.08-1.96) and surgery from HVS (OR=1.55; 95% CI: 1.10-2.17) had higher odds of having ≥2 CEA tests (Table 3). Compared to no CRT, receiving neoadjuvant CRT plus adjuvant chemotherapy (OR=5.27; 95% CI: 3.47-8.00), neoadjuvant CRT (OR=2.65; 95% CI: 1.81-3.87) and adjuvant CRT (OR=3.75; 95% CI: 2.12-6.61) were associated with ≥2 CEA tests.

### CT tests

As shown in Table 1, 209 (20%) patients had ≥2 CTs (56% had ≥1). The median months to first image was 5 (IQR=2- 8). The median number of CTs for the entire cohort and those who had ≥2 CT tests was 1 (IQR=0-1) and 2 (IQR=2-2), respectively. In bivariate analyses, younger age, female gender, being married, lower comorbidity, lower function-related indicator and any CRT sequence (vs none) were associated with ≥2 CTs (Table 2).

Younger age (66-70 vs > 80 years, OR=1.78; 95% CI: 1.10-2.89; >70-75 vs > 80 years, OR=2.12; 95% CI: 1.33-3.39) was associated with associated with ≥2 CTs (Table 3). Compared to no CRT, receiving adjuvant CRT (OR=6.88; 95% CI: 3.60-13.14), neoadjuvant CRT plus adjuvant chemotherapy (OR=4.20; 95% CI: 2.50-7.01) and neoadjuvant CRT (OR=2.79; 95% CI: 1.67-4.67) had higher odds of ≥2 CTs.

### Sensitivity analyses

The results of the various sensitivity analysis are not shown. In a sensitivity analysis excluding 204 patients with ‘indicated’ colonoscopies, 43% (n=356) of patients received ≥1 colonoscopy and surgery from HVS (OR=1.64; 95% CI: 1.11- 2.43) was significantly associated with ≥1 colonoscopy (not significant in main analysis). Compared to the main analysis, a sensitivity analysis restricting physician visits to oncologists and colorectal surgeon specialists had similar post-treatment surveillance utilization rate (42%) and identical significant predictors of ≥2 physician visits.

Given that the goal of surveillance is to detect recurrence, we did a sensitivity analysis in which recurrent patients were included in the study and considered adherent; median months to recurrence was 5 (IQR=3-10) and the recalculated surveillance rates increased to 71% for ≥1 colonoscopy, 71% for ≥2 physician visits, 66% for ≥2 CEA tests and 50% for ≥2 CTs. In another sensitivity analysis excluding patients aged 80+, surveillance rates increased to 64%, 60%, 53% and 24% for colonoscopies, physician visits, CEA tests and ≥2 CTs, respectively.

## Discussion

Among the 1,024 patients with stage II/III rectal cancer, receipt of guideline-recommended surveillance colonoscopy (55%), physician visits (54%), CEA tests (47%) and CTs (20%) was low at 14 months post-treatment. However, since a higher percentage of these patients received at least one physician visit (85%), CEA test (66%) and CT (56%) within the recommended timeline, this suggests that most patients initiate but do not complete post-treatment surveillance. The reasons for this phenomenon are unclear but previous studies have suggested that lack of patient self-management tools, patient preferences and failure to adequately communicate with patient play a role in receipt of guideline-recommended surveillance [31,32].

A key finding consistent with previous research is that younger age was significantly associated with receipt of all four guideline-recommended post-treatment surveillance measures [33]. The low surveillance among older patients is indicative of the clinical dilemma physicians face given the heterogeneity in physiological fitness among older patients that makes it challenging to achieve consensus onthe risk-benefit of surveillance [33]. Higher incidence of side-effects from primary rectal cancer treatment among patients aged 80+ years may account for the low surveillance rates in older patients.33 It is possible that patient preferences or perceived risk-benefit by either the patient or physician may account for lower surveillance rates among older patients [33,34]. While clinical factors, such as comorbidities and functional impairment, could explain lower surveillance among older patients, after adjusting for multiple factors, those clinical factors were not significant predictors of receipt of any surveillance procedures in this study. Higher risk pathology or healthy-adherer bias may be driving the association between receipt of CRT and post-treatment surveillance.

Patient demographic characteristics and surgeon volume were not consistently significant across all surveillance procedures; this suggests that the weight of these factors in the decision to receive surveillance varies by surveillance procedure. Being married was associated with having guideline-recommended colonoscopies, physician visits and CEA tests likely because it offers psychosocial support, greater economic and logistical access [35,36]. Females had higher receipt of physician visits and CEA tests; the reason for this remains speculative but variation in gender preferences to cancer care [37] may explain this finding. The significant association between urban status and CEA test adherence may suggest variation in practice patterns by rurality as reported previously [38]. The significance of drive time to nearest HVH (colonoscopy model) and race (physician visits model) suggest the significance of access to quality care in surveillance receipt [20,39]. The higher odds of physician visits and CEA tests among HVSs suggest that surgeons play a major role in post-treatment surveillance referrals [40] this is more apparent in the absence of an imperative to seek care on the patient’s part by the significance of HVS in the sensitivity analysis of colonoscopies without indications (i.e., HVS not significant in main model including ‘indicated’ colonoscopies).

This study has several limitations such as lack of information on physician recommendations, patient preferences and reasons for surveillance. While lack of information on reasons for surveillance procedures could mean that physician visits could be for non-cancer surveillance reasons, the similarity in results between the main analysis and sensitivity analysis restricting physician visits to oncologists and colorectal surgeon specialists suggests the extent of this is minimal. As published previously [19] the identification of recurrence based partly on receipt of treatment for the recurrence may not capture patients who decided to forgo treatment resulting in under-estimated surveillance rates for patients who opted out of recurrent cancer treatment, or an overestimation of surveillance if procedures were performed due to suspected recurrence. To minimize the impact of this, we excluded patients whose adjuvant treatment was >6 months or those who sought hospice treatment before or during the surveillance period. Similar to the algorithm by Deshpande et al. [19] which identified 18.4% of recurrent cancer, our study identified 16.7% rectal cancer recurrences. This published algorithm was reported to have 81% sensitivity and 99% specificity,19 supporting that our algorithm correctly excluded cases it detected as recurrent but under-identified them. While this could explain lower surveillance rates among patients aged 80+, exclusion of this population showed marginally higher but still sub-optimal surveillance rates. Even though an argument can be made that exclusion of recurrent patients may underestimate surveillance, the sensitivity analyses including recurrent cancer patients showed slightly higher but sub- optimal surveillance rates. Furthermore, since the median of 5 months to recurrence diagnosis suggests tests for recurrent patients were for diagnostic versus surveillance purposes, this supports the argument to exclude recurrent patients. Despite these limitations, the post-treatment surveillance estimates in this study are similar to recent studies [41,42] and add relevant knowledge to the ongoing problem of sub-optimal surveillance by describing factors associated with surveillance.

## Conclusion

In conclusion, our study indicates sub-optimal post-treatment surveillance. This is a significant public health challenge given the high rectal cancer recurrence rates and sub-optimal survival outcomes [4,43]. Although associations with age and CRT treatment were relatively consistent across guideline-recommended surveillance procedures, associations with patient characteristics and surgeon volume were specific to particular surveillance procedures suggesting that multicomponent interventions may be necessary to increase post-treatment surveillance uptake. Future studies should determine the types of multicomponent interventions that can bridge the gap between guideline-recommended care and real-world challenges to receipt of post-treatment surveillance.

## Figures and Tables

**Figure 2a:**
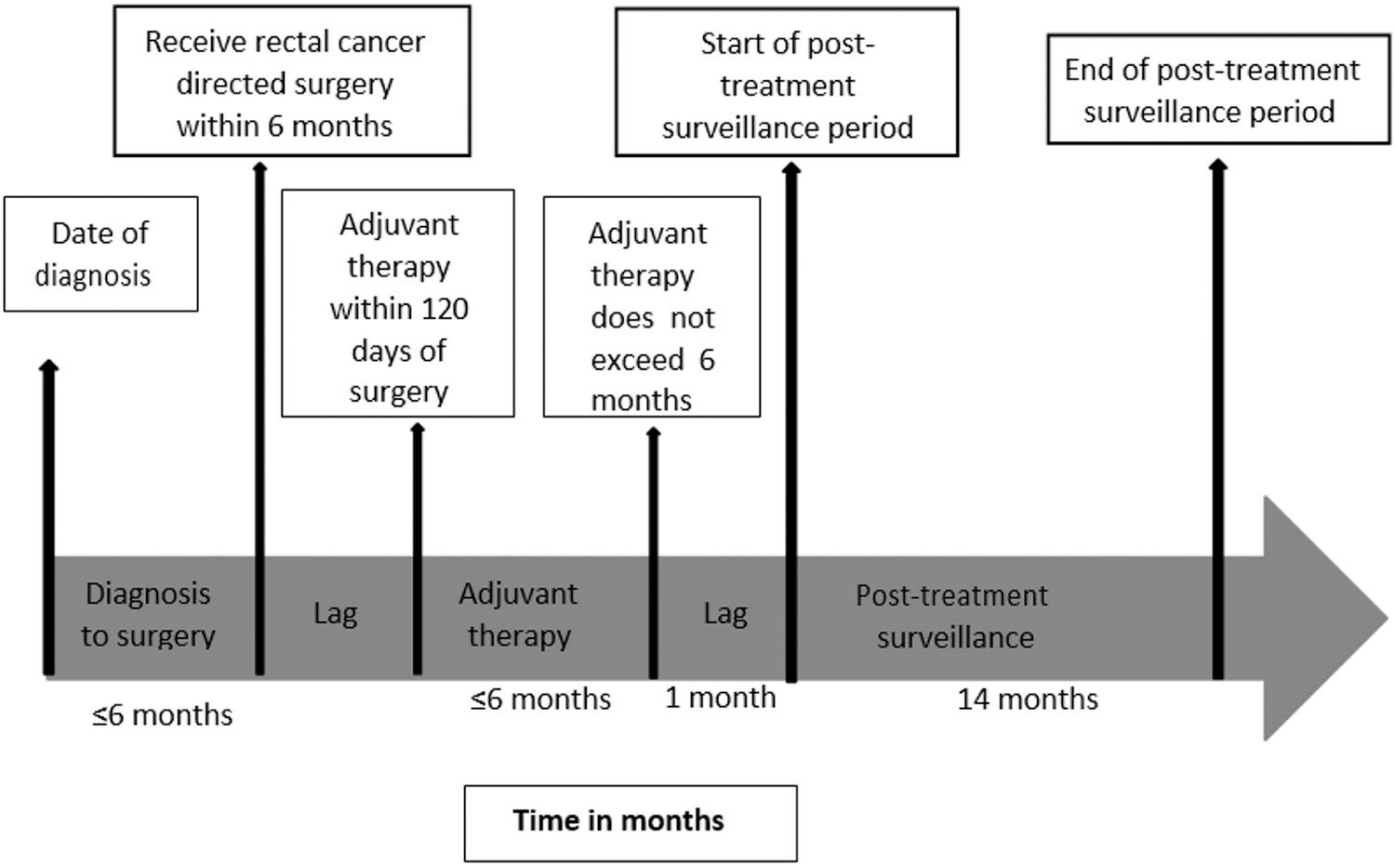
Post treatment surveillance timeline for stage II/III rectal adenocarcinoma patients who had adjuvant therapy.

**Figure 2b:**
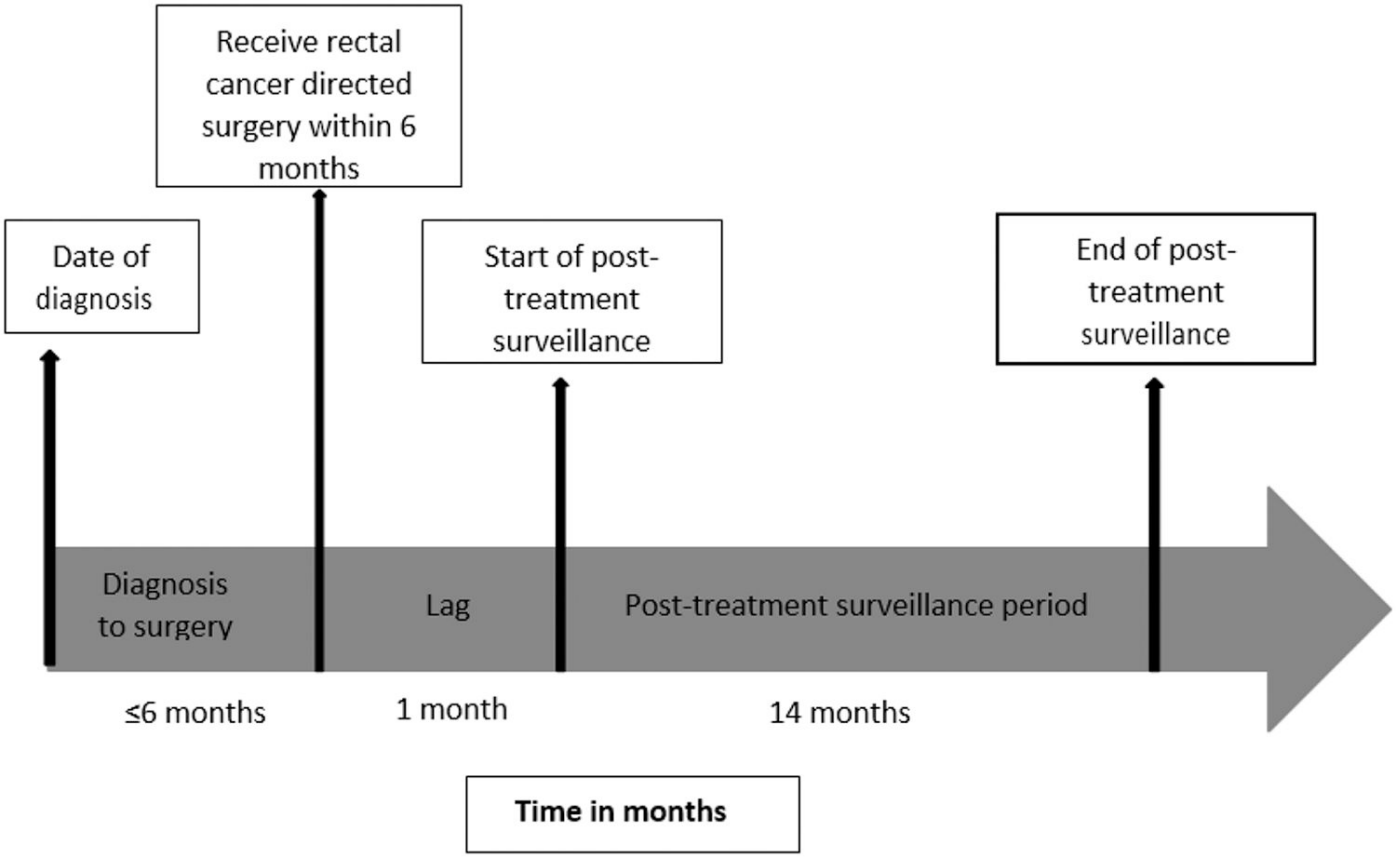
Post-treatment surveillance timeline for stage II/III rectal adenocarcinoma patients who did not have adjuvant therapy.

**Table 1:** Number of surveillance tests and months to first surveillance procedure for stage II/III rectal adenocarcinoma patients during the 14-month post-treatment surveillance period.

Characteristic			Percentiles				
Colonoscopy	Patient Population	N (%)	Median (IQR)	Minimum	10^th^	90^th^	Maximum
Surveillance procedure	All Patients	1024 (100%)	1 (0-1)	0	0	2	4
	Patients had ≥1 colonoscopy	560 (55%)	1 (1-2)	1	1	2	4
Months to 1^st^ colonoscopy	Patients had ≥1 colonoscopy	560 (55%)	5 (3-9)	1	2	12	15
Physician visit							
Surveillance procedure	All Patients	1024 (100%)	2 (1-2)	0	0	3	5
	Patients had ≥2 physician	552 (54%)	2 (2-3)	2	2	4	5
Months to 1st physician	Patients had ≥1 physician	867 (85%)	2 (1-4)	1	1	6	15
CEA tests							
Surveillance procedure	All Patients	1024 (100%)	1 (0-2)	0	0	3	5
	Patients had ≥2 CEA tests	478 (47%)	3 (2-3)	2	2	4	5
Months to 1^st^ CEA test	Patients had ≥1 CEA tests	677 (66%)	4 (2-6)	1	1	9	15
CT							
Surveillance procedure	All Patients	1024 (100%)	1 (0-1)	0	0	2	4
	Patients had ≥2 CT	209 (20%)	2 (2-2)	2	2	3	4
Months to 1st CT	Patients had ≥1 CT	573 (56%)	5 (2-8)	1	1	12	15

**Table 2:** Characteristics of stage II/III rectal adenocarcinoma patients by receipt of guideline-recommended surveillance procedures during the 14-month post-treatment surveillance period, row %.

		Received ≥1 Colonoscopy			Received ≥2 Physician				Received ≥2 CEA test			Received ≥2 CT tests		
Characteristic		N (%)	No n=464	Yes n=560	P-value	No n=472	Yes n=552	P-value	No n=546	Yes n=478	P-value	No n=815	Yes n=209	P-value
Age	66-70	241 (24)	32	68	<0.0001	42.7	57.3	<0.0001	46.5	53.5	<0.0001	72.2	27.8	<0.0001
	>70-75	229 (22)	38.4	61.6		38.4	61.6		41.9	58.1		70.3	29.7	
	>75-80	216 (21)	37.5	62.5		39.8	60.2		51.8	48.2		84.7	15.3	
	>80	338 (33)	64.5	35.5		57.7	42.3		66.9	33.1		87.9	12.1	
Gender	Male	549 (54)	46.7	53.3	0.3944	44.4	55.6	0.3179	52.8	47.2	0.7754	82.5	17.5	0.0301
	Female	475 (46)	44.1	55.9		47.5	52.5		53.7	46.3		77	23	
Marital status	Not married^1^	511 (50)	53.6	46.4	<0.0001	52.6	47.4	<0.0001	60.3	39.7	<0.0001	83	17	0.0073
	Married	513 (50)	37	63		39.6	60.4		46.4	53.6		76.2	23.8	
Race	Other/Unknown	148 (14)	50	50	0.2155	56.8	43.2	0.0049	57.4	42.6	0.2783	82.4	17.6	0.3536
	White	876 (86)	44.5	55.5		44.3	55.7		52.6	47.4		79.1	20.9	
Rural status	Rural	221 (22)	48.4	51.6	0.2952	45.7	54.3	0.6331	52.3	47.7	0.214	80.5	19.5	0.1939
	Urban^1^	803 (78)	44.5	55.5		47.5	52.5		57	43		76.5	23.5	
Living below	Above median	489 (48)	47	53	0.2898	49.5	50.5	0.0372	56.2	43.8	0.0737	80.6	19.4	0.4557
poverty indicator	Below median	535 (52)	43.7	56.3		43	57		50.7	49.3		78.7	21.3	
With college	Above median	540 (53)	43.9	56.1	0.3337	46.1	53.9	0.9906	52.8	47.2	0.7132	79.6	20.4	0.9734
Education	Below median	484 (47)	46.9	53.1		46.1	53.9		53.9	46.1		79.6	20.4	
Stage	II	598 (58)	45.1	54.9	0.9018	45.3	54.7	0.555	53.7	46.3	0.7852	78.8	21.2	0.4634
	III	426 (42)	45.5	54.5		47.2	52.8		52.8	47.2		80.8	19.2	
Charlson score	0	543 (53)	41.2	58.8	0.0009	42.5	57.5	0.006	48.2	51.8	<0.0001	77.3	22.7	0.0464
	1	274 (27)	44.9	55.1		46	54		52.9	47.1		79.6	20.4	
	2+	207 (20)	56.5	43.5		55.6	44.4	0.0378	67.1	32.9		85.5	14.5	
Function-Related	0	665 (65)	42.6	57.4	0.0016	43.8	56.2		50.3	49.7	<0.0001	77	23	0.0212
indicator	1	211 (21)	44.3	55.7		46.7	53.3		53.8	46.2		84.3	15.7	
	2+	148 (14)	58.8	41.2		55.4	44.6	<0.0001	66.2	33.8		84.5	15.5	
CRT sequence received	No CRT	360 (25)	57.2	42.8	<0.0001	59.4	40.6		73.1	26.9	<0.0001	91.7	8.3	<0.0001
	Neoadjuvant CRT + Adjuvant Chemo	211 (27)	31.8	68.2		30.8	69.2		29.9	70.1		67.8	32.2	
	Neoadjuvant CRT	226 (25)	44.7	55.3		42.9	57.1		47.8	52.2		77	23	
	Adjuvant CRT only	69 (8)	23.2	76.8		36.2	63.8		40.6	59.4		62.3	37.7	
	Other	158 (15)	46.8	53.2		44.9	55.1		53.2	46.8		79.1	20.9	
Surgical	No	706 (69)	43.1	56.9	0.0309	42.6	57.4	0.0009	49.3	50.7	0.0001	79	21	0.513
complications	Yes	318 (31)	50.3	49.7		53.8	46.2		62.3	37.7		80.8	19.2	
Surgeon volume^2^	High volume	631 (62)	41.7	58.3	0.012	41	59	<0.0001	47.2	52.8	<0.0001	78	22	0.2657
	Low volume	286 (28)	50.7	49.3		52.1	47.9		61.5	38.5		82.2	17.8	
	Unknown	107 (10)	52.3	47.7		59.8	40.2		67.3	32.7		82.2	17.8	
Hospital volume^2^	High volume	637 (62)	41.3	58.7	0.0032	44.3	55.7	0.2753	49.6	50.4	0.0059	78.3	21.7	0.3526
	Low volume	243 (24)	50.6	49.4		50.2	49.8		61.3	38.7		82.7	17.3	
	Unknown	144 (14)	54.2	45.8		47.2	52.8		56.3	43.7		79.9	20.1	
Proximity to	0-15 minutes	277 (27)	37.2	62.8		46.9	53.1		53.8	46.2		80.5	19.5	
high-volume	>15-30 minutes	169 (16)	49.7	50.3	0.0096	49.7	50.3	0.6823	55.6	44.4	0.9019	85.2	14.8	0.1608
hospital	30+ minutes	293 (29)	45.7	54.3		45.1	54.9		52.6	47.4		76.8	23.2	
	Unknown	285 (28)	50.2	49.8		44.2	55.8		52.3	47.7		78.3	21.7	

**Table 3:** Association between stage II/III rectal adenocarcinoma patient characteristics and receipt of ≥1 colonoscopy, ≥2 physician visit, ≥2 CEA test and ≥2 CT tests during the 14- month post-treatment surveillance period, Odds ratio (95% Confidence Interval).

Characteristic		N (%)	Adjusted Odds* of ≥1 Colonoscopy	P-value	Adjusted Odds* of ≥2 Physician Visits	P-value	Adjusted Odds* of ≥2 CEA Tests	P-value	Adjusted Odds* of ≥2 CT Tests	P-Value
Age	66-70	241 (24)	3.23 (2.18-4.78)	<0.0001	1.30 (0.89-1.91)	0.0069	1.35 (0.91-2.00)	0.028	1.78 (1.10-2.89)	0.0013
	>70-75	229 (22)	2.48 (1.70-3.62)		1.61 (1.10-2.34)		1.81 (1.23-2.66)		2.12 (1.33-3.39)	
	>75-80	216 (21)	2.58 (1.77-3.77)		1.57 (1.08-2.28)		1.24 (0.84-1.81)		0.98 (0.58-1.66)	
	>80	338 (33)	Ref.		Ref.		Ref.		Ref.	
Gender	Male	549 (54)	Ref.	0.1153	Ref.	0.0034	Ref.	0.0145	Ref.	0.4688
	Female	475 (46)	1.27 (0.94-1.70)		1.56 (1.17-2.09)		1.45 (1.08-1.95)		0.88 (0.62-1.25)	
Marital status	Not married^1^	511 (50)	Ref.	0.0005	Ref.	0.0339	Ref.	0.0128	Ref.	0.3195
	Married	513 (50)	1.69 (1.26-2.26)		1.56 (1.17-2.08)		1.46 (1.08-1.96)		1.28 (0.79-2.08)	
Race	Other/Unknown	148 (14)	Ref.	0.0817	Ref.	0.0276	Ref.	0.231	Ref.	0.7403
	White	876 (86)	1.41 (0.96-2.08)		1.79 (1.23-2.62)		1.27 (0.86-1.88)		1.06 (0.75-1.51)	
Rural status	Rural	221 (22)	Ref.	0.6626	Ref.	0.8579	Ref.	0.1275	Ref.	0.3099
	Urban^1^	803 (78)	1.09 (0.73-1.64)		1.20 (0.81-1.79)		1.38 (0.91-2.09)		0.78 (0.48-1.26)	
Living below	Above median	489 (48)	1.05 (0.76-1.45)	0.7767	0.79 (0.57-1.08)	0.1404	0.88 (0.63-1.22)	0.4478	0.84 (0.57-1.25)	0.3901
poverty indicator	Below median	535 (52)	Ref.		Ref.		Ref.		Ref.	
With college	Above median	540 (53)	1.09 (0.73-1.64)	0.6268	0.82 (0.59-1.14)	0.0753	0.87 (0.62-1.22)	0.4242	1.03 (0.68-1.55)	0.9027
education	Below median	484 (47)	Ref.		Ref.		Ref.		Ref.	
Stage	II	598 (58)	1.11 (0.84-1.47)	0.4552	1.27 (0.97-1.66)	0.1596	1.18 (0.89-1.56)	0.258	1.37 (0.97-1.92)	0.0708
	III	426 (42)	Ref.		Ref.		Ref.		Ref.	
Charlson score	0	543 (53)	1.32 (0.91-1.92)	0.3403	1.18 (0.82-1.70)	0.5721	1.47 (1.00-2.17)	0.1318	1.30 (0.80-2.13)	0.521
	1	274 (27)	1.22 (0.81-1.82)		1.16 (0.78-1.73)		1.42 (0.94-2.17)		1.12 (0.66-1.90)	
	2+	207 (20)	Ref.		Ref.		Ref.		Ref.	
Function-	0	665 (65)	1.30 (0.86-1.96)	0.3393	1.13 (0.76-1.69)	0.486	1.31 (0.85-2.01)	0.4726	1.18 (0.69-2.01)	0.204
indicator	1	211 (21)	1.41 (0.88-2.24)		1.07 (0.68-1.69)		1.23 (0.76-2.00)		0.80 (0.43-1.48)	
	2+	148 (14)	Ref.		Ref.		Ref.		Ref.	
CRT sequence received	No CRT	360 (25)	Ref.	0.0001	Ref.	<0.0001	Ref.	<0.0001	Ref.	<0.0001
	Neoadjuvant CRT + Adjuvant Chemo	211 (27)	1.90 (1.27-2.85)		2.71 (1.81-4.06)		5.27 (3.47-8.00)		4.20 (2.50-7.01)	
	Neoadjuvant CRT	226 (25)	1.30 (0.89-1.89)		1.70 (1.17-2.45)		2.65 (1.81-3.87)		2.79 (1.67-4.67)	
	Adjuvant CRT only	69 (8)	3.97 (2.11-7.47)		2.41 (1.37-4.24)		3.75 (2.12-6.61)		6.88 (3.60-13.14)	
	Other	158 (15)	1.28 (0.85-1.94)		1.76 (1.17-2.65)		2.32 (1.52-3.52)		2.77 (1.58-4.85)	
Surgical	No	706 (69)	Ref.	0.6143	Ref.	0.3095	Ref.	0.3347	Ref.	0.0906
complications	Yes	318 (31)	1.08 (0.80-1.47)		0.85 (0.63-1.14)		0.86 (0.63-1.17)		1.38 (0.95-2.02)	
Surgeon volume^2^	High volume	631 (62)	1.22 (0.87-1.70)	0.3722	1.47 (1.06-2.04)	0.0163	1.55 (1.10-2.17)	0.0011	1.21 (0.80-1.84)	0.5455
	Low volume	286 (28)	0.95 (0.58-1.56)		0.73 (0.45-1.18)		0.71 (0.43-1.20)		0.95 (0.50-1.79)	
	Unknown	107 (10)	Ref.		Ref.		Ref.		Ref.	
Hospital volume^2^	High volume	637 (62)	1.18 (0.82-1.70)	0.3661	1.04 (0.73-1.49)	0.1469	1.33 (0.92-1.94)	0.1026	1.32 (0.83-2.11)	0.3307
	Low volume	243 (24)	0.83 (0.48-1.45)		0.79 (0.46-1.36)		0.81 (0.46-1.42)		0.92 (0.47-1.82)	
	Unknown	144 (14)	Ref.		Ref.		Ref.		Ref.	
Proximity to	0-15 minutes	277 (27)	1.56 (1.00-2.43)	0.0388	1.03 (0.67-1.59)	0.709	0.86 (0.55-1.34)	0.674	0.92 (0.55-1.55)	0.366
high-volume	>15-30 minutes	169 (16)	0.88 (0.55-1.41)		1.00 (0.63-1.61)		0.88 (0.54-1.42)		0.64 (0.35-1.16)	
hospital	30+ minutes	293 (29)	Ref.		Ref.)		Ref.		Ref.	
	Unknown	285 (28)	0.99 (0.62-1.56)		1.28 (0.81-2.02)		1.13 (0.71-1.81)		1.10 (0.65-1.87))	

## Table A1: Codes and SEER-Medicare and Rural-Urban commuting area (RUCA) files used to identify primary and recurrent rectal cancer treatment, recurrent cancer diagnosis, diagnostic indications for colonoscopy and rural status.

**Table:**

	Codes	Files
Rectal Cancer-Directed Surgery based on Hierarchy and Combination		
Used to identify receipt of rectal cancer surgery	CPT: 44145, 44146, 44147, 44155-44156, 44157-44158, 44207-44208, 44209, 44211, 44212, 44238, 44239, 45110, 45111-45116, 45119-45120, 45123, 45126, 45160, 45170-45172, 45190, 45395, 45397, 45499, 45999 ICD-9 Procedure: 45.75-45.76, 45.94-45.95, 46.03, 46.1, 46.10-46.13, 46.2, 46.20- 46.23, 48.3, 48.31-48.36, 48.4, 48.40-48.43, 48.49, 48.5, 48.50-48.52, 48.59, 48.6, 48.60, 48.61, 48.63-48.65, 48.69, 48.7, 48.70-48.76, 48.79-48.82, 48.90-48.93, 48.99 SEER Surgery Codes: 10-14, 20-25, 28, 30, 40, 50, 60, 80, 90 (primary rectal cancer surgery only)	NCH, MEDPAR, PEDSF, Outpatient
**Adjuvant or Neoadjuvant Therapy**		
Chemotherapy	CPT/HCPC (Agents): Any chemotherapeutic agents in the C-, G-, J-, Q-, S- series ICD-9 Procedure: 99.25, 99.28, 00.10 ICD-9 Diagnosis: V58.1, V58.11, V58.12, V66.2, V67.2 NDC Codes: Corresponding to capecitabine or any other oral chemotherapeutic agent	NCH, Outpatient, DME, HHA, MEDPAR, Part D
Radiation Therapy	CPT: 77261-77299, 77300-77381, 77399, 77400-77499, 77520- 77525, 77600- 77620, 77750-77799 HCPC: C1715-C1720, C2616, C2632-C2643, C2698, C2699, C9725, C9728, D5983- D5985, Q3001, S8049 ICD-9 Procedure: 92.21-92.29	NCH, Outpatient, MEDPAR
**Rectal Cancer Recurrence**		
Used to identify rectal cancer recurrence	ICD-9 Diagnosis: 196.0-196.3, 196.7, 197, 197.0-197.8, 198.0-198.8, 198.81, 198.82, 198.89, 199.0, 199.1	NCH, Outpatient, MEDPAR
**Diagnostic Indication**		
Used to identify diagnostic indications for colonoscopy	ICD-9 Diagnosis: Anemia: 280.0, 280.1, 280.8, 280.9, 281.0- 281.4, 281.8, 281.9, 285.0, 285.1, 285.2, 285.9; Gastrointestinal bleeding: 286.5, 459.0, 562.02, 562.03, 562.12, 562.13, 569.3, 569.84-569.86, 578.1, 578.9, 792.1, 998.11; Constipation: 560.1, 560.81, 560.89, 560.9, 564.0, 564.00, 564.09, 564.01, 564.02; Diarrhea: 008.42, 008.43, 008.45, 008.5, 008.8, 009.0–009.3, 558.1-558.3, 558.9, 564.4, 564.5, 564.7- 564.9, 787.91, 078.5; Abdominal pain: 789.0, 787.3, 789.4, 789.6; Ischemic bowel disease: 557.0, 557.1, 557.9; Bowel habits change or Incontinence of feces: 787.99, 787.6; Fistula: 565, 569.81, 596.1; Hemorrhoids: 566, 455; Secondary cancer or suspicion of metastasis: 150–2, 155–9, 162–5, 170–6, 179–199; Diverticulitis, Radiation colitis, Volvulus: 562.11, 558.1, 560.2; Impaction of intestine: 560.30, 560.39; Abnormal radiology of gastrointestinal tract: 793; Weight loss or Protein calorie malnutrition;783.2; 783.3, 783.4, 260–263; Stenosis of rectum and anus: 569.2; Ulcer: 569.41, 569.82; Colostomy or anastomosis complications: 569.6, V44.3, V45.3, V55.3, 997.4; Dermatomyositis: 710.3; Injury or foreign body in colon and rectum,: 863.4, 936	NCH, Outpatient, MEDPAR
**Rural Status**		
Rural	RUCA codes: 3.0, 4.0, 5.0, 6.0, 7.0, 7.2, 7.3, 7.4, 8.0, 8.2, 8.3, 8.4, 9.0, 9.1, 9.2, 10.0, 10.2, 10.3, 10.4, and 10.5	2010 RUCA file
Urban	RUCA codes: 1.0, 1.1, 2.0, 2.1, 3.0, 4.1, 5.1, 7.1, 8.1, 10.1	

## Abbreviations:

CEA: Carcinoembryonic Antigen Tests
Chemo: Chemotherapy
CI: Confidence Interval
CMS: Centers for Medicare and Medicaid Services
CPT: Current Procedural Terminology
CRT: Chemoradiation Therapy
CT: Computed Tomographic Colonography
DME: Durable Medical Equipment
FRI: Function-Related Indicator
HHA: Home Health Agency
HVH: High-Volume Hospital
HVS: High-Volume Surgeon
ICD_9: International Classification of Diseases, Ninth Revision Clinical Modification
MEDPAR: Medicare Provider Analysis and Review
NCCN: National Comprehensive Cancer Network
NCH: National Claims History
NCI: National Cancer Institute
OR: Odds Ratio
PEDSF: Patient Entitlement and Diagnosis Summary File
RUCA: Rural-Urban Commuting Area Classification
SEER: Surveillance Epidemiology and End Results
TME: Total Mesorectal Excision
US: United States of America
